# Rural self-reliance: the impact on health experiences of people living with type II diabetes in rural Queensland, Australia

**DOI:** 10.3402/qhw.v9.24182

**Published:** 2014-06-24

**Authors:** Althea Page-Carruth, Carol Windsor, Michele Clark

**Affiliations:** Faculty of health, Queensland University of Technology, Brisbane, Australia

**Keywords:** Rural, culture, type II diabetes, chronic disease, Australia, interpretive, grounded theory

## Abstract

**Objective:**

The objective of the study was to explore whether and how rural culture influences type II diabetes management and to better understand the social processes that rural people construct in coping with diabetes and its complications. In particular, the study aimed to analyse the interface and interactions between rural people with type II diabetes and the Australian health care system, and to develop a theoretical understanding that reflects constructs that may be more broadly applicable.

**Methods:**

The study applied constructivist grounded theory methods within an interpretive interactionist framework. Data from 39 semi-structured interviews with rural and urban type II diabetes patients and a mix of rural health care providers were analysed to develop a theoretical understanding of the social processes that define diabetes management in that context.

**Results:**

The analysis suggests that although type II diabetes imposes limitations that require adjustment and adaptation, these processes are actively negotiated by rural people within the environmental context to fit the salient social understandings of autonomy and self-reliance. Thus, people normalized self-reliant diabetes management behaviours because this was congruent with the rural culture. Factors that informed the actions of normalization were relationships between participants and health care professionals, support, and access to individual resources.

**Conclusions:**

The findings point to ways in which rural self-reliance is conceived as the primary strategy of diabetes management. People face the paradox of engaging with a health care system that at the same time maximizes individual responsibility for health and minimizes the social support by which individuals manage the condition. The emphasis on self-reliance gives some legitimacy to a lack of prevention and chronic care services. Success of diabetes management behaviours is, however, contingent on relative resources. Where there is good primary care, there develops a number of downstream effects including a sense of empowerment to manage difficult rural environmental circumstances. This has particular bearing on health outcomes for people with fewer resources.

Although Australia has the fourth most urbanized population in the world (Organization for Economic Development, 2009), 12% of the population live in outer regional or remote areas (Australian Bureau of Statistics, 2010; Australian Institute of Health and Welfare, 2008). However, the experiences, beliefs, and cumulative health encounters of rural people with regard to health are relatively under-researched aspects of the chronic disease experience.

Chronic disease accounts for 80% of the total disease burden in Australia and its management accounts for 70% of all current health expenditure (Australian Institute of Health and Welfare, 2006), a high proportion of which is directed to diabetes because of long-term treatment and symptomatic complications. Because 92% of costs associated with diabetes are attributable to type II diabetes (T2DM) (Begg et al., 2007) and because T2DM cuts across many aspects of the health care system, it was considered an appropriate condition through which to view and analyse peoples’ health experiences.

The purpose of this study was to better understand how Australian people living in rural areas of southern Queensland experience a chronic disease and to develop a theoretical appreciation of rural behaviours. In particular, we sought to explore the core social processes that underpin the management of T2DM and its health complications within the context of rural Australia and the rural health care system.

The Australian health care system (AHCS) is a complex mix of public and private services administered by different levels of government and across government and non-government sectors. There is a noted disparity in the number of health professionals between metro and remote areas and, in the latter areas, limited access to specialist services (Commonwealth of Australia, Rural Health Standing Council, 2012). Furthermore, rural populations have more health risk factors and a higher mortality rate related to diabetes than those in the urban areas (Australian Institute of Health and Welfare, 2010). Much of the variation between metro and remote health status has been explained in terms of socio-economic factors (Turrell, Kavanagh, & Subramanianc, 2006). This paper presents the results of a study that examined how the Australian rural culture shapes the management of T2DM and rural people’s interactions with the AHCS and concludes that the rural culture itself exacerbates rural or urban disparities.

## Methodology and research methods

The theoretical framework underpinning the study was grounded in the philosophies of symbolic interactionism and identity theory (Blumer, 1962, 1969; Burke & Stets, 2009; Mead, 1934; Stryker, 1968, 1980) and the cultural approaches of Kityama, Duffy and Uchida (2007) and Markus and Hamedani (2007). The works of Berger and Luckmann (1966), Bourdieu (1996, 1999), and Giddens (1984) added a contextual dimension to the analysis of human behaviours.

From the premises of the above works, we understand that human beings construct their worlds through experience and that meaning and emotional responses are mediated through people’s actions and reactions to the actions of others (Blumer, 1969, p. 4). As such, a life-time of social contexts are interpreted, re-interpreted, and selected by the individual to become social, role, and person identities (Burke & Stets, 2009). The greater the commitment to and salience of a particular identity, the more likely those meanings are absorbed and perceived to be personally relevant. As patterns of conduct are internalized and taken for granted, they shape consciousness and subsequent health behaviours. It is argued here that experiences within the Queensland rural and remote environment create internalized meanings and expectations of rural culture, particularly around self-reliant and responsible behaviour.

The study applied both shared and unique features of grounded theory methods inherent to the constructivist approach of Charmaz (2003, 2006). These incorporated theoretical sampling and simultaneous collection and analysis of data, the constant comparative analysis of data, and a focus on the development of theory (Charmaz, 2006). The process saw the application of theoretical questions as ideas developed (Charmaz, 2006, 2008) rather than imposing structured theoretical concepts (Glaser, 1978, 1992, 1998). These techniques provided a way of synthesizing data so that developing concepts established a connection between theory and the empirical world.

The intent of the research was to sample and interview those with knowledge and experience of T2DM (Glaser, 1978; Morse, 1991; Morse, Barrett, Mayan, Olson, & Spiers, 2002), that is, people who could provide information about life with diabetes, in all its dimensions, including interactions with the AHCS. Initial sampling had to proceed along purposeful lines as the researcher had no evolving theory to act as a guide (Cutcliffe, 2000). The concept of theoretical sampling (Glaser & Strauss, 1967) was adopted as the analysis evolved but sampling commenced with “criterion sampling” which invited people with T2DM, who were over the age of 18 and living in Queensland in the area defined by the Australian Standard Geographical Classification (ASGC) Remoteness Areas (Australian Institute of Health and Welfare, 2004). The “outer regional,” “remote,” and “very remote” descriptors of the ASGC encompass approximately 12% of Australia’s population and accurately cover the geographical area relevant to this study (Australian Bureau of Statistics, 2011). Remoteness is measured in terms of physical distance by road to an urban centre (Australian Institute of Health and Welfare, 2004).

Ethical approval was granted by the District Health and Queensland University of Technology Human Research Ethics Committees. Other approvals were obtained, as part of the process of theoretical sampling, from the Queensland Health Department and the Royal Australian College of General Practitioners. The relevant health districts provided a list of names and addresses of all people who had presented to a rural hospital within the previous 12 months with T2DM. Potential participants were those with T2DM living in or near towns with a population of no more than 5000 people. This group was contacted by letter and asked to participate. For these people, community health services were variable and the nearest tertiary health services were in Toowoomba which was situated between 200 and 1500 km away from their place of residence.

Fourteen people from 230 invitations responded initially, but as analysis progressed it was deemed important to include rural people who had experienced management of T2DM only within the primary health sector. Equally, the perspectives of people living in an urban context were deemed valuable in exploring the concept of self-reliance and its relationship to personal support mechanisms and support by health care practitioners (HCPs) and the AHCS. The latter sample group contributed to an exploration of preventative health behaviours and any differences between the urban and rural contexts in how people used services. Both these groups self-referred on the basis of information provided in the waiting rooms of GP practices in various locations.

An additional source was HCPs who delivered services to rural people with T2DM in the identified rural area. A wide group of rural HCPs was contacted and individuals also self-referred. The details of the final sample appear in Table I.

**Table I:** Summary of participant sample.

Number of rural people with T2DM interviewed in ASGC areas 3–5	19
Number of urban people with T2DM interviewed	5
Number of HCPs interviewed including one person who had T2DM but who was interviewed specifically in their capacity as a HCP	8
Number of rural people without diabetes interviewed	4
Number of rural people with T2DM interviewed a second time	3

The first author undertook 39 interviews with 36 participants and the data generated was sufficiently rich to develop significant theoretical outcomes. Each interview was approximately 1 h long and were undertaken in participant homes or workplaces. In the latter part of the study, three individuals who had been interviewed previously and were at ease with the interviewing process were invited to be re-interviewed to further explore the rural culture. The people with diabetes who were interviewed constituted a diverse group of individuals with varying health experiences. There were 19 rural and 5 urban participants with diabetes. These participants had received their diagnoses between 3 months and 30 years prior to interview; ages ranged from 33 to 88; rural men outnumbered women, whereas it was the opposite in the urban settings. The sample included two Indigenous persons with diabetes, one of whom was an Aboriginal care worker. Other health care professionals interviewed included a general practitioner, podiatrist, dietician, diabetes educator, diabetes educator/practice nurse, diabetes service administrator, and a community manager. Four people without diabetes from different rural backgrounds were also interviewed to provide more insight into the rural culture and rural health services in general. The participants varied in ages (28–80) and lived in either outer regional or more remote and very remote areas. All came from English-speaking backgrounds and had varying degrees of health knowledge.

Data analysis commenced directly following the first interview, and constant comparison of data and categories was conducted through an iterative process of initial, focused, and theoretical coding and a return to the raw data (Charmaz, 2006). Memos and journals recorded evolving analytical ideas that were used throughout the study.

During the initial phase, interview data were coded line-by-line. This resulted in the identification of concepts developed from participant stories of the experiences of rural life, the management of diabetes, and interactions with the AHCS. The analytical significance of the concepts was further explored in subsequent interviews and through relevant literature. Ultimately in comparing data with data in a constant comparison process, it was possible to develop focused codes. All data were then compared to the focused codes which were refined into interrelated subcategories. At one stage of the study, these subcategories were considered as continuums of participant behaviours (Table II).

**Table II:** An example of subcategories at one stage of the study.

< --------------------------- Belonging -------------------------- >
< ---------------------- Being supported ---------------------- >
< ------------------------------- Coping ----------------------------- >
< -------------------- Gaining mastery/control -------------------- >
< ---------------------- Gaining self-confidence ---------------------- >
< ---------------------- Developing independence ---------------------- >
< ---------------------- Being hopeful and optimistic ---------------------- >

As the study progressed, the cognitive components became subsumed within a broader framework where individual participant voices represented processes shared with others. The dominant concepts were robust and persistent over repeated coding although the codes were provisional in the sense that they were modified as data collection and analysis continued until a core category was generated. The criteria for establishing the core category was that it was central, that it related to as many categories and properties as possible, and that it accounted for a large portion of the variation in the data (Holton, 2007). The analysis explored how these concepts related to one another and underpinned an overarching theoretical explanation of how rural culture shapes the management of T2DM. This moved the process to a condensed abstract view where the scope and dimension of the analysis moved beyond pure description.

## Results

**Figure 1:**
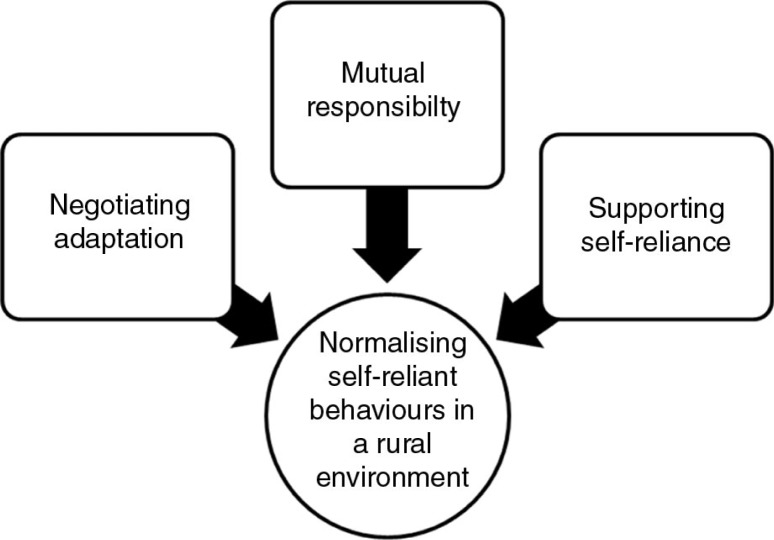
The relationship between theoretical codes.

*Normalizing self-reliant behaviours in a rural environment* was the core category that explained relationships between the concepts noted in Figure 1 as participants set about managing their T2DM. This was not a term used by any one participant but reflects the totality of actions of the rural participants as the chronic disease imposed limitations on everyday life. Thus, normalizing self-management captured the sociological process of living with T2DM in a context that demanded behaviours take account of the self-reliant cultural framework and experience of rural people in southern Queensland.

The concept of normalization depicted a process whereby people constructed meanings about their lives and then acted upon those constructions. They minimized struggles and adjustments, became optimistic about the prognosis, redefined the normal state as the present level of functioning, reordered priorities and values, sought information that validated personal experiences, and engaged in favourable comparisons with others who were worse off. Meanings, however, assumed a particular form within cultural contexts and thus shaped the normalization process. Hence, the rural participants with diabetes acted in ways that maintained identities applicable to the rural environment and reinforced the structure of care and resources in the rural society within which they lived.

Normalization is always a construction because it involves making choices or discriminating between experiences within a social context. In this sense, a hierarchy of social, role, and person identities (Burke & Stets, 2009) came to define and verify the normalization process. This meant that once the rural participants came to terms with the diagnosis of T2DM, they used existing social and personal identities to normalize the diabetes into role identities, that is, they demonstrated the personal and social identity concepts of self-reliance, self-determination, and self-efficacy and integrated these personal and social identities into the self-management tasks.

The rural environment required that participants normalize self-reliant behaviours to fit the Australia rural context to the point that T2DM became absorbed into daily activities and, ultimately, appeared inconsequential. This participant’s comment was indicative:

I just lead a normal life. I just take my insulin with me in my little dilly bag and whack it in whosever’s fridge I’m at or keep it in a little case it comes in with its little cool brick, yeah. It doesn’t affect me at all. And when I go to have it, I have it. (Lily ASGC 4)

Thus, experiences were actively reconstructed to support the self-reliant identity. Behaviours were gradually modified in order to align perceptions with an identity standard and this affected the way rural participants presented to others. This was not “denial” as interpreted by Kelleher (1988), rather the concept of self-reliance functioned as a reference point for appraising a fit between agentic identities and diabetes management objectives (Ahearn, 2001).

Between the tablets and the diet, I just keep doing, what they prescribed and it just keeps everything going, flows along nicely. (Pete ASGC 4)

Even when confronted with what they saw as permanent non-modifiable outcomes of diabetes, such as Rob who had a leg amputated, this did not “diminish self-care” as suggested by Rubin (2000). In these cases, the focus was on altering the emotions (feelings) stimulated by the situation. It did not change the self-view as self-reliant but it reinforced the identities of integrity and resilience. Participants simply accepted that negative situations had to be borne.

## Negotiating adaptation

The contributing concept of *negotiating adaptation* explained that, over time, the condition imposed limitations that required adaptation but within the rural environment the level of adaptation was negotiated to match cultural norms and conditions. For some the process of adaptation was slower as they did not, at first, perceive that life with T2DM warranted action. The realization came, however, that even when there were no overt symptoms, self-management behaviours maintained better function and independence. Personal health needs were not always fully understood and participants did not always adapt in a perfect clinical sense, but adaptation was framed to fit the expectations of their peers and their own social circumstances. For example, as a rural participant explained:

I just go about my normal ways and I’m feeling well, I haven’t changed too much, other than I watch, try to limit particularly sugars. I’m not into drinking a lot of soft drink or anything. If I drink a bit of scotch I have soda water with it, plenty of ice and I’ve just watched sugary things, other than fruit I won’t go without that. (Sam ASGC 5)

Autonomy and control over diabetes was explicit in the way diabetes management for rural participants was approached. In the words of one participant:

Unless you take control of the disease for yourself and manage your health and manage the impact that the diabetes has on you, it will control you … Options just aren’t available out here. (Mat ASGC 3)

Acceptance of responsibility and a belief that success would follow action therefore converged with a rural context that demanded self-reliance.

Brought up in the bush all my life … you know there’s a need for certain things. (Sam ASGC 5)

As a consequence, rural participants with diabetes rarely brought expectations of greater help to the notice of HCPs and nor did they demand access to the variety and number of services accessed by urban people. Instead, participants accepted the need to mitigate the risks attributed to poor diabetes control but did so without being fully informed and supported at the various stages of diabetes prognosis.

An associated factor was the delivery of services and support by the AHCS to rural areas that was seen by many to be a low priority, as is made clear in this comment:

[I] Asked if they had any literature that they could give me, I had nothing here and there were no support groups, there was nothing … “Go home and go on a diet.” What diet? Only that my mother and I had been used to it with dad. No help. Absolutely no help at all. (Dot ASGC 4)

Budgetary constraints on rural services compared to those in urban areas (Philips, 2009) reinforce an expectation on the part of service providers that the rural culture of self-reliance can itself be relied upon to compensate for poorer chronic care and preventative health services. Diabetes activities are expected to proceed relatively unsupervised and unaided and therefore challenges and problem-solving are aligned with the cultural framework. Access to minimal tools, information, and resources reinforces the self-reliance of rural people in diabetes management and commits them to a self-reliant social identity.

The limited amount of support received from HCPs and the AHCS compared to those in the urban context provided a notable contrast. This catalogue of urban services was typical:

My last appointment up there when I saw the young man, he said, “Would you like to see our practice nurse to have a diabetes management plan?” And I said, Why would I need that? And he said, Oh, well, it might help. And I said, Hang on, my blood sugars have been good for ages, I have a podiatrist, I have a physio, I have an endocrinologist and I see a cardiologist and I see a rheumatologist, and none of them seem to have issues so why do I need to see a practice nurse? I said, it’s gotten bigger than a string ensemble, it needs a conductor! (Judy ASGC 1)

Rural people did not question the lack of support but a few participants with greater resources, who understood the value of regular checks with optometrists and podiatrists for example, actively sought out information further afield to manage diabetes more effectively. A participant noted:

Yes that was another thing I just did off my own bat. [The doctor] didn’t sort of tell me to do it. It was just through, I suppose reading diabetes stuff. I noticed almost immediately that I started on the Diabex and things my eyesight went right off again … The other thing I’d chased up was the podiatrist …. They both said basically yeah, “You’ve got your baseline, we don’t know where you’re at so we can monitor what happens if it’s happened and if you’re improving or getting worse or whatever. (Bill ASGC 5)

Situating responsibility for diabetes management with individuals has therefore become the primary pathway of T2DM management in rural areas in the absence of services that are elsewhere the accepted standard of care (Colagiuri, Dickinson, & Girgis, 2009; Diabetes Australia & Royal Australian College of General Practitioners, 2011). In rural areas, the discourse of self-reliance had become the rationalization for a lack of service provision to a point where participants did not demand preventative or chronic care services but rather focused on the availability of emergency assistance:

I do expect the ambulance to come out here and pick me up if I’m really sick, but only if I’m really sick. (Bob ASGC 3)

Visiting a busy primary rural HCP was justified in acute situations but seeing a HCP for a check on a chronic condition could be delayed. A rural HCP put this succinctly:

You know that doctors are only to be seen when you are sick and not when you’re not sick. (Betty ASGC 5 HCP)

Unless the diabetes deteriorated suddenly, in which case this constituted an emergency, the rural participants relegated it a lesser priority while continuing to believe they were managing the diabetes responsibly. When asked about the significance of diabetes in their lives, rural responses were similar to this participant:

No, I wasn’t overly worried, no. I don’t know why not. (Kris ASGC 4)

Although there were some appropriate preventative services available to rural people with diabetes, the services were not always consistent and could not be relied upon. A HCP described the situation in the following terms:

I think you’ve got to realise that there are distances involved and they don’t have equal access to allied health, and allied health is fine, but it’s not always available and we’re talking about dieticians coming every 2 weeks. But there’s been periods of time when there’s no incumbent in a position and no one comes for, you know, 6 weeks. There’s not the access that you get to all the range of allied health that you would in a metropolitan area. (Neli ASGC 4 HCP)

Variability of opportunity meant referrals were not always encouraged by the primary HCPs and both HCPs and rural participants accepted this approach to diabetes management. Consequently, participants remained both unaware of services and undemanding and few were encouraged to access other services such as diabetes physicians, optometrists, podiatrists, or other multi-disciplinary programs:

The GP’s attitude was, “Well, you used to be a nurse back then, you look after your foot yourself. You don’t need all these other people to help you.” Because that’s what the GP said, this person then declined other services. (Cath ASGC 4 HCP)

The combination of being deprived access to diabetes rural support networks, ignorance of services or information, and the complexity of the AHCS structure makes it more difficult for people to navigate and retrieve the broader diabetes management processes that are available. Many are therefore not recipients of practical and other support. This situation is rarely challenged but is taken for granted by rural people because of years of experiencing poor health care delivery.

Rather, people internalize responsibility for diabetes self-management because it matches the actions of HCPs, the structure of the AHCS, and accords with the rural self-reliant identity. At the same time, a culture is created where individuals are seen as responsible for poor BG results or adverse consequences. A HCP summarized the majority view:

Very frequently, they will be told that the reason their diabetes is out of control is because there are some lifestyle changes that they should engage in. (Stan ASGC 5 HCP)

When people’s homeostatic glycaemic function deteriorates naturally, many HCPs viewed individuals more negatively and critical normative judgments were imposed on those who failed to display “approved” levels of self-efficacy, responsibility, and self-reliance, as reflected in this comment:

… some people, like no matter what you do, they won’t comply. But people that are, you know, motivated, they will have some effect and the allied health input, umm, they’ll take it up and they’ll run with it and, but if you’re not motivated. (Neli ASGC 4 HCP)

Reflexively, the rural AHCS has capitalized on the rural self-reliant culture and positioned itself as a resource to be used largely in acute emergency situations and is structured accordingly. People with T2DM assume a self-reliant identity congruent with the expectations of the AHCS and HCPs and all parties collude with the view that when the diabetes deteriorates sufficiently there is always access to tertiary services. Rural people are, as a result, less attuned to mutual engagement in information transfer, team care assessments, and referral opportunities within the primary care system (Australian Ministers Advisory Council’s National Rural Health Policy Sub-Committee & National Rural Health Alliance, 2002; Commonwealth of Australia, Rural Health Standing Council, 2012). They seek to make sense of the condition through the expression of self-reliant actions until they can no longer manage, at which time they turn to hospital services. The result is that rural people receive poorer preventative and chronic health care services not only because of the structure of the AHCS but because they reproduce and endorse the system and the behaviours of HCPs.

## Mutual responsibility

Where the relationship between the practitioner and the individual did demonstrate commitment and shared responsibility, better care was perceived by the rural participants. This *mutual responsibility* informed actions and relationships with HCPs and in these cases rural people gained greater confidence in and commitment to self-managing diabetes practices.

It wasn’t until I spoke to the diabetes educator; she went through it and then spoke to the dietician. The dietician then, lovely lady, helped me unbelievably. Then I went up to Clifton to work for a contractor, his mother was a district nurse out at Millmerran. Living two weeks with her was …. I couldn’t have done it. She told me exactly what I had to do and between them all it put my life back on track. (Mat ASGC 3)

Although the rural SES is more heterogeneous than the urban environment (Australian Institute of Health and Welfare, 2008, 2010), not all rural people have the same capabilities even though they are subject to the same self-reliant identity constraints. As such, where a culture demands self-reliance in circumstances of chronic conditions such as T2DM, the focus is on self-management. Depending on self-management without direction, however, does not ensure positive clinical outcomes.

Where HCPs conveyed respect for the capacity of people to take responsibility and at the same time assumed a shared responsibility to manage BG levels, a self-reliant identity was reinforced. This relational connection between individuals, HCPs, and the AHCS was therefore crucial to rural people’s diabetes outcomes. In an environment where there are relatively few primary care services, only those with greater access to resources have the opportunity to overcome conflicting pressures and to achieve more positive diabetes outcomes.

## Supporting self-reliance

The impact of access to resources, whether financial, institutional, or relational, was seen to be critical. The concept of *supporting self-reliance* refers to the value of sustaining self-management behaviours and how the structure of the AHCS and actions of HCPs influenced participants’ self-concepts during the health interaction process. Those with supportive relationships developed a greater sense-of-self as independent and had more options available to maintain identity commitments. In the case below, there was some financial support facilitated by a mutually supportive HCP:

I’m re-pat you see, I’m a veteran and re-pat did all the worrying for me so I didn’t worry. They joined me up with the diabetes people … I don’t have to pay for anything, they did the whole bit. They sent me one of those little bags with the testing strips. They send me fresh things to put them in. (Jan ASGC 3)

Others with fewer resources were compelled to accept that which was available and were unwilling to act in any way that might put the HCP–patient relationship at risk. For example, Lily related well to a HCP in another town, but the clinic was 160 Km away:

I’d quite easily change to him, but it’s the hassle of, we’ve got an hour’s drive over and an hour’s drive home, and then the hassle of, can you get an appointment. (Lily ASGC 4)

Low-income families have to accept whatever local services are offered because they are unable to access more distant HCPs (Strasser, Harvey, & Burley, 1994; Wathen & Harris, 2007). Where there are few choices, positive and negative HCP–patient relationships become disproportionately influential. Those with less health knowledge or poorer relationships delay appropriate help-seeking action and compound the impression of poor compliance. In such circumstances, participants with T2DM gained little from health interactions and assumed that the role of the independent self-reliant individual meant just that.

Pre-existing resource distribution therefore operates as a source of power and serves as a basis by which meanings are constructed and reproduced. Participants with greater access to resources were able to take control and manage diabetes in line with opportunities. Having resources enabled some to manifest self-reliant identities such as initiating self-help groups while others met challenges by accessing advice and support further afield. Those unable to mobilize resources learnt that much of what they encountered was beyond their control. As has been argued elsewhere (Heine, 2007), the optimal way to maintain independence was to emphasize integrity and resilience during difficult times. Rural people with T2DM therefore adhere to a cultural framework of greater individual responsibility and accept that the fault for poor BG results, slow progress, or adverse consequences lies with them.

The irony was that rural participants who experienced more support perceived themselves as more self-sufficient in diabetes management. Support enhanced self-confidence and encouraged the search for better knowledge. Hence, where there was good primary care, this resulted in a number of flow-on effects. It allowed for the normalization of diabetes in ways congruent with the context while encouraging participants to increase their understanding of health and to direct self-reliance towards positive clinical outcomes. It provided an environment for education and good self-management and a sense of empowerment to manage difficult contextual circumstances. As such, support contributed to the often spoken about rural self-reliance and self-sufficiency albeit in a mutually interdependent process.

## Conclusions

The term rural is not, as Pong, DesMeules, and Lagace (2009) note, an undifferentiated entity. Nonetheless, a rural cultural framework of self-sufficiency, independence, self-reliance, and the subsequent normalization of T2DM self-management actions is built upon the reality of rural living and distance from supportive institutional structures. Self-reliance is not only expected of rural people but is also a feature of the capacity to live and work in an environment with fewer support services. The social and personal self-reliant identities (Burke & Stets, 2009) are intrinsic to approaches to on-going diabetes management and also to relationships with rural HCPs and the AHCS. Few people expect support and most are socialized to take responsibility.

It is because diabetes is positively sensitive to glycaemic control through diet, exercise, or medication, that the rural self-reliant identity is compatible with diabetes self-management as a concept. It is viewed through the same cultural framework that shapes other rural behaviours (Bourdieu, 1996, 1999; Giddens, 1984; Kityama, Duffy, & Uchida, 2007; Markus & Hamedani, 2007), and in the absence of individualized information, people seek to resolve health issues by drawing upon cultural practices and rural self-reliant social norms to manage the condition. People reflexively monitor actions, then normalize and re-create personal diabetes self-management behaviours which conform to the self-reliant cultural framework and context of rural society.

Rural diabetes management behaviours are therefore a product of cultural, social, political, and economic settings that have accumulated over years of rural socialization. This research suggests that rural self-reliance is conceived as the primary strategy of chronic diabetes management and the emphasis on self-reliance has given some legitimacy to a lack of prevention and chronic care service provision. In short, people with T2DM face the paradox of engaging with a system that maximizes individual responsibility for health and at the same time minimizes the social support by which individuals manage the condition.

Rural people come to interpret diabetes management within the constraints of the rural environment and this is contingent on resources. Resources create choices and the potential to mediate rural structural constraints. The rural environment thus structures the conditions for self-reliance but also the context for poorer clinical outcomes. Reinforcing a self-reliant identity in the absence of adequate resources contributes to stress and the likelihood that an individual will seek to use services only when diabetes causes serious complications. Hence, the framework of individual responsibility and self-reliance engenders an acceptance of responsibility for poorer outcomes. It is concluded therefore that the concept of self-management needs to be interpreted within context and its meaning not assumed to be politically, culturally, and socially neutral.

## Study limitations

In interpretive research, there are limits to the extent of generalizations that can be made beyond the group of study participants. The intent here, however, was to develop an account of the theoretical dimensions of the experiences of a participant group within a defined context and at a particular historical point in time. Nonetheless, the findings may have broader applicability as inequities in the provision of health resources and health status between urban and outer regional and remote areas is a, if not uniform, global phenomenon.
